# Notes and News

**Published:** 1892-04-23

**Authors:** 


### Notes and News.

Collected and Communicated.

We are glad to hear that a convalescent home for women, the wives and daughters of artisans, and for those earning their own livelihood in the metropolis, is about to be started before long. It seems strange that such an establishment should not have existed before now, especially as one for men has been provided for some time. It is proposed that the home should be situated at the seaside, and that, except in very urgent cases, payment should be required. The inmates of the men's home, which is at Dover, pay from 10s. to 12s. a week, sums which many can afford for a short period, and which render the institution nearly self-supporting. The Committee of the new home consists of ladies, and if they prove as energetic as the ladies of Birmingham in finding friends to help, as in the case of the children's home, the new institution will soon be established.

The friends of the North-West London Hospital are setting an excellent example. At the opening of the year the financial condition of the institution was very grave, and it appeared necessary to curtail the sphere of useful work. To avoid this an appeal was made, which met with prompt response, Baron de Hirsch heading the subscription list with £1,000, and Mr. John Maple's offer of £100, provided twenty others contributed £50 each, secured the required sums. The Scotch saying that "Ilka little makes a muckle" was verified by the results of Mr. Beckwith Smith's half-crown fund. The North-West Hospital has several admirable charitable schemes in connection with it. The "George Sturge Samaritan Fund," which provides nourishment and change of air for convalescents, assisted twenty-four patients for the moderate expenditure of £33 8s. These schemes which act as an extension of hospital relief are more beneficial than yet fully recognised, and were their powers greater the return of patients to the hospitals owing to relapse would be as rare as it is now frequent. Such an institution as the soup kitchen, which is established during the winter in the neighbourhood of the hospital, is an excellent thing for out-patients; 14,190 of these were under treatment at the hospital during the past year, during which time 646 in-patients received admission.

A well-known contemporary of ours, in enumerating a variety of amusements for Easter, remarks that "there are more ways than one of spending a holiday." This thought may have inspired advertisers in the same journal to wild efforts of emulation as to novelty, for the following is offered us as an Easter Monday programme in another column:—

> Only three more Easters remain—in 1893, 1894, and 1895—before Christ's Second Advent in the air to translate 144,000 living Christians to heaven without dying, on March 5th, 1896, prior to His descent at Jerusalem on the last day of this age, April 11th, 1901. Several ministers will explain these and other prophetic truths to-day (Easter Monday) at meetings at three and seven o'clock, in Beaumont Hall, Beaumont Square, Mile-end Road, East London; and next Sunday in Kensington Town Hall, at eleven, three, and seven o'clock. The greatest European war ever known is expected soon this year, according to Daniel.

Doctors with nervous patients will, we expect, reap a harvest after this!

When the difficulties of site are considered, it is not surprising that the need for fever hospitals exceeds the supply. The would-be establishers of such an institution almost invariably bring at least one hornets' nest about their ears. The proposal made at the last meeting of the Asylums Board is agitating North London in the neighbourhood of Highbury and Clissold Park. The promoters of the new fever hospital scheme desire the healthiest locality they can find for their establishment, but the inhabitants of the healthy neighbourhood conjure a hot-bed of infection to be planted in their midst, and very naturally resist the invasion. The hospital is invited to occupy a still more salubrious resting-place further from the largely inhabited districts right away in the country, and, as the only site suggested was inordinately expensive, it is possible, Highbury will have its wish. At the same meeting at which the obnoxious scheme was proposed, the excellence of isolation hospitals on board ship, especially in small-pox cases, was advocated. Small-pox has appeared more frequently in some districts than should be owing, it is said, to laxity in carrying out the Compulsory Vaccination Acts by some of the authorities.

It is now finally decided, as the result of the last meeting of the Court of Contributors of the Edinburgh Royal Infirmary, that a sum of £100,000 is to be expended on the extension of the hospital. The difficulty of site is overcome, and the additional buildings will occupy the ground on which stood the Sick Children's Hospital, and where stands still one of the schools of the Merchant Company. For this property it has been arranged that the Infirmary shall pay the sum of £12,000. The plans now placed before the authorities provide a large pavilion at a southern end of the ground, parallel and similar to the existing medical pavilions, to contain 84 beds. Two pavilions run east and west, one of three storeys, and the other of two, to contain 60 and 40 beds respectively; and there is one small pavilion of one story, near the present surgical ward, farthest west, to contain 12 beds. The space available affords ample means of ventilation and lighting, and the plans have met with general approval; £100,000 is, however, a large sum to raise, but hope seems to prevail that the amount will ultimately be forthcoming. Edinburgh will be leaving the metropolis far behind in the race, as without this extra provision, the number of beds to population already exceeds that of London.

